# 7.0 Tesla MRI Brain Atlas: *In vivo* atlas with cryomacrotome correlation

**Published:** 2010-04-07

**Authors:** Antonio A. F. De Salles, Alessandra A. Gorgulho

**Affiliations:** 1 Department of Neurosurgery, David Geffen School of Medicine, University of California, Los Angeles, California-90095, USA; 2 Department of Radiation Oncology, David Geffen School of Medicine, University of California, Los Angeles, California-90095, USA

**Figure:**
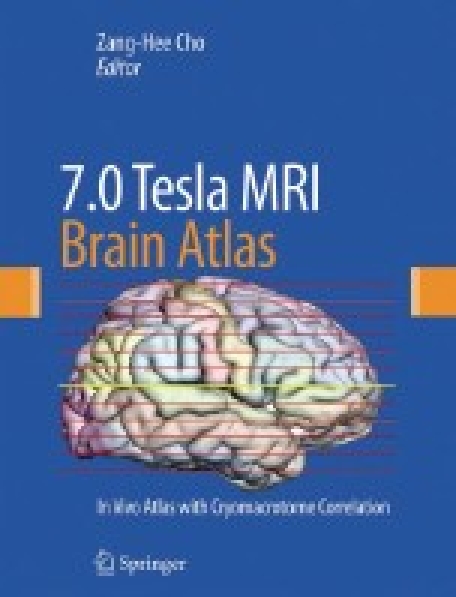


This is a monumental work combining neuroanatomy and neuroradiology. Outstanding neuroscientists came together under Dr. Zang-hee Cho leadership, a neuroscientist from Gachon University of Medicine and Science, to develop this Korean groundbreaking work. Their product promises to be the new reference atlas for functional neurosurgeons and neuroscientists at large. They took advantage of the developments of anatomy and imaging through the 20^th^ century, setting stage for the exciting capabilities of ultra high field (UHF) magnetic resonance imaging (MRI).

The methods of structure localization the authors applied match that of stereotactic surgery, which has been the core of the scientific understanding of the human brain physiology, allowing at this time the burgeoning field of neuromodulation. This field progressed in the end of the last century with the imaging revolution brought about by computed images. After its establishment for treatment of chronic pain and movement disorders, it is currently being extended to Gilles de la Tourette syndrome, major depression and obsessive-compulsive disorder. As these and other exciting applications such as non-focal epilepsy and morbid obesity enter neurosurgical practice, the importance of detailed anatomical knowledge encompassing a broader gamut of brain regions is core.

This atlas brings knowledge from exquisite anatomy specimen preparation matched to modern radiological imaging techniques. Its reach becomes even broader as imaging merging capabilities now allow the digital form of this compendium to be combined to stereotactic localization software widely available in the clinical world. The well designed and consistent methodology of this atlas allied to the exquisite attention to details is timely to permit the development of one of the most promising and elegant fields in neurosurgery, neuromodulation, which undoubtedly depends on such reliable anatomy to exploit functional imaging.

Cadaveric and UHF-MRI slices are referenced and compared based on anterior and posterior commissural planes. Planes are centered in the mid-commissural point, therefore adopting standard Cartesian coordinates used in stereotactic surgery. This makes this 7.0 Tesla MRI Atlas of great relevance for stereotactic surgeons. At 2 mm intervals, the images are compared taking advantage of various magnifications to detail the *in vivo* visualization of the brain structure. The bulk of the representation is however radiological-anatomical comparison 1 to 1, i.e. true size. The authors have tried to comply with the most common standard of MRI studies in the western world, i.e. the viewer sees the brain images as if seeing axial slices caudal to cranial bound and coronal slices front to back bound, therefore right side is on the left hand of the viewer on axial and coronal images. Such simple details make this work easy to use as a reference for neuroradiologists and neurosurgeons alike.

At most fine detail, as atlases are used in stereotactic surgery, common targets such as subthalamic nucleus, globus pallidus internun and thalamic nuclei, although represented in this atlas, neurosurgeons will still have to rely on traditional stereotactic measurements in their individual patient, as we have yet to have a reliable probabilistic atlas capable to obviate the need for electrophysiology and traditional stereotactic approaches. These measurements are well represented by Cho et al assuring their work usefulness during the years to come. This atlas is a must in the library of functional neurosurgeons.

